# Impact of Susceptibility Testing Methodology on the Positioning of Cefiderocol and Aztreonam-Avibactam Against Metallo-β-Lactamase-Producing Gram-Negative Bacteria

**DOI:** 10.3390/antibiotics15040380

**Published:** 2026-04-09

**Authors:** Fernando del Nogal-Labrador, Beatriz González-Blanco, María Isabel Sanz, Raúl Recio, Patricia Brañas, Irene Muñoz-Gallego, Esther Viedma, Jennifer Villa

**Affiliations:** 1 Department of Clinical Microbiology, Hospital Universitario 12 de Octubre, 28041 Madrid, Spain; 2 Instituto de Investigación Sanitaria, Hospital Universitario 12 de Octubre (imas12), 28041 Madrid, Spain

**Keywords:** cefiderocol, aztreonam-avibactam, metallo-β-lactamase, antimicrobial resistance, antimicrobial susceptibility testing, EUCAST, broth microdilution

## Abstract

**Background/Objectives:** The impact of antimicrobial susceptibility testing methodology on the categorization and positioning of cefiderocol and aztreonam-avibactam against metallo-β-lactamase (MBL)-producing Gram-negative bacilli remains unclear. This study aimed to evaluate the in vitro activity of cefiderocol and aztreonam-avibactam against clinical MBL-producing isolates and to assess the agreement between different cefiderocol susceptibility testing methods. **Methods:** A total of 299 non-duplicate clinical MBL-producing Gram-negative isolates were collected from clinical samples between 2022 and 2025. Antimicrobial susceptibility testing was performed using broth microdilution, disc diffusion, and gradient strip diffusion according to European Committee on Antimicrobial Susceptibility Testing (EUCAST) criteria. Carbapenemase genes were identified by immunochromatography and multiplex PCR. Categorical agreement and error rates between cefiderocol testing methods were analyzed. **Results:** *Klebsiella pneumoniae* was the predominant species, mainly producing NDM alone or in combination with OXA-48-like carbapenemases. Aztreonam-avibactam demonstrated complete activity against all Enterobacterales isolates (262/262, 100%) and high activity against *Pseudomonas* spp. (33/37, 89%). Cefiderocol susceptibility among Enterobacterales varied markedly depending on the testing method. Disc diffusion yielded 14% susceptibility (37/262), which increased to 52% (136/262) after ATU resolution, whereas broth microdilution showed 85% susceptibility (224/262). This resulted in low categorical agreement (42%) and a high rate of major errors (58%), with no very major errors detected. Cefiderocol activity did not differ substantially across carbapenemase types and was highest against VIM-producing *Pseudomonas* spp. **Conclusions:** Aztreonam-avibactam showed consistent in vitro activity against MBL-producing Enterobacterales, whereas cefiderocol activity was strongly influenced by the susceptibility testing methodology. Disc diffusion substantially underestimated cefiderocol susceptibility compared with broth microdilution. These findings highlight the critical impact of testing methodology on cefiderocol categorization and support the therapeutic role of last-line agents in the management of MBL-producing Gram-negative infections, with direct implications for clinical microbiology laboratories and antimicrobial stewardship programs.

## 1. Introduction

Carbapenemase-producing Gram-negative bacilli (GNB) represent a major global health threat due to limited therapeutic options and significant infection-related mortality [1]. The global burden of carbapenem resistance continues to rise, with attributable deaths increasing from approximately 600,000 in 1990 to over 1 million in 2021 [2]. In the latest World Health Organization (WHO) Bacterial Priority Pathogen List, carbapenem-resistant Enterobacterales remain classified as Critical Priority pathogens, *Klebsiella pneumoniae* ranking the highest, while carbapenem-resistant *Pseudomonas aeruginosa* has recently been reclassified as High Priority [3].

Metallo-β-lactamases (MBLs) are particularly worrisome because of their broad hydrolytic spectrum and the lack of therapeutic options. Within this group, New Delhi metallo-β-lactamases (NDM) have shown a rapid global dissemination and are increasingly implicated in hospital outbreaks [4]. In Spain, OXA-48-like carbapenemases remain predominant among Enterobacterales, followed by KPC carbapenemases, whereas NDM carbapenemase types still account for fewer than 10% of isolates [5]. Although OXA-48-like carbapenemases remain the most frequently detected among Enterobacterales in our institution, a marked increase in NDM-producing isolates has been observed in recent years, making NDM the second most prevalent carbapenemase type and the dominant MBL in our setting.

Two new antimicrobial agents have recently expanded therapeutic options against MBL-producing GNB, cefiderocol, a siderophore cephalosporin [6], and aztreonam-avibactam, both recommended as first-line options for these infections in the latest Infectious Diseases Society of America (IDSA) guidelines [7]. Recent studies have demonstrated that cefiderocol exhibits potent activity against MBL-producing organisms [8]; however, substantial variability in reported susceptibility rates has been observed depending on the testing methodology and interpretative criteria applied, raising concerns regarding the comparability and reliability of results across laboratories [9]. In addition, emerging resistance to both antibiotics has already been reported, highlighting the need for continuous surveillance to track resistance evolution and support evidence-based antimicrobial stewardship [10,11].

In this context, the impact of susceptibility testing methodology on the categorization and clinical positioning of cefiderocol remains insufficiently characterized, particularly in real-world settings with a high prevalence of NDM-producing isolates. Given the increasing incidence of MBL-producing Enterobacterales in our institution, our setting provides a relevant opportunity to assess these methodological discrepancies under conditions of sustained selective pressure. Therefore, to contribute to the optimization of antimicrobial resistance management, we aimed to characterize susceptibility patterns and evaluate how testing methodology influences the interpretation and positioning of cefiderocol and aztreonam-avibactam in a high-NDM-prevalence setting.

## 2. Results

### 2.1. Bacterial Isolates

A total of 299 MBL-producing GNB clinical isolates from individual patients were included. Urinary tract samples were the most common source (n = 129, 43%), followed by skin and soft tissue infections (n = 61, 20%), respiratory tract samples (49, 16%), blood cultures (n = 47, 16%), and intra-abdominal infections (n = 13, 4%). Most isolates originated from medical wards (n = 196, 66%), whereas 65 (22%) were recovered from surgical units and 34 (12%) from intensive care units.

*K. pneumoniae* was the most prevalent species (n = 221, 74%), followed by *P. aeruginosa* (n = 27, 9%) and *Enterobacter cloacae* complex (n = 16, 5%). The remaining 35 isolates (12%) comprised *Escherichia coli*, *Citrobacter freundii*, *Klebsiella aerogenes*, *Klebsiella oxytoca*, *Klebsiella variicola*, *Serratia marcescens*, and *Pseudomonas* spp. (*Pseudomonas moselii*, *Pseudomonas monteilii*, and *Pseudomonas putida*).

**Figure 1:**
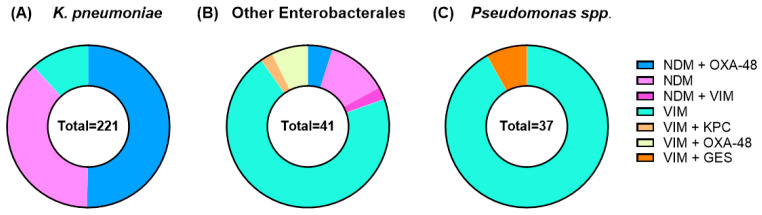
Distribution of carbapenemases among clinical MBL-producing isolates. Donut charts show the proportion of carbapenemases identified in (**A**) *K. pneumoniae* (n = 221), (**B**) other Enterobacterales (n = 41), which comprised *E. coli*, *C. freundii*, *K. aerogenes*, *K. oxytoca*, *K. variicola,* and *S. marcescens*, and (**C**) *Pseudomonas* spp. (n = 37).

Among Enterobacterales, 113 isolates (43%) co-produced NDM and OXA-48-like carbapenemases, 89 (34%) produced NDM alone, 55 (21%) VIM, 3 VIM/OXA-48-like, 1 VIM/KPC, and 1 VIM/NDM co-detections. Among *Pseudomonas* spp., 34 (92%) carried VIM and 3 (8%) co-produced VIM and GES carbapenemases. The distribution of MBL types is illustrated in Figure 1.

### 2.2. Antimicrobial Susceptibility Testing

#### 2.2.1. MBL-Producing Enterobacterales

The MIC_50_ and MIC_90_ values for all antibiotics tested are shown in Table 1. All Enterobacterales isolates exhibited a multidrug-resistant phenotype, with high resistance rates to meropenem (193/262; 74%). Among the classic antibiotics, only amikacin and gentamicin retained consistent activity, with MIC_50_ values below their respective clinical breakpoints, while colistin showed an MIC_90_ within the susceptibility range. Stratification by carbapenemase revealed comparable susceptibility profiles among NDM/OXA-48-like co-producers and the overall cohort, although slightly lower aminoglycoside MICs were observed in the former. Among isolates producing NDM alone, colistin was the only traditional agent active against more than 50% of isolates. Conversely, VIM-producing Enterobacterales demonstrated a more favorable and heterogeneous profile, with susceptibility rates of 87% to meropenem, 100% to amikacin, and 89% to colistin.

**Figure 2:**
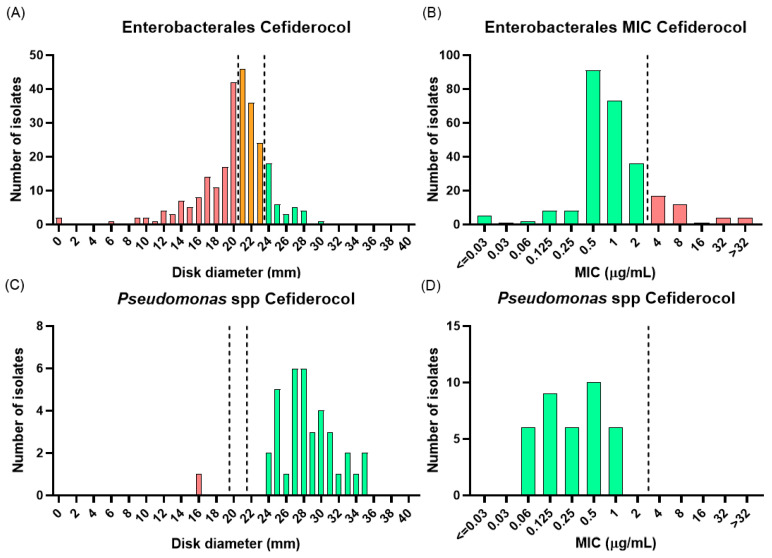
Cefiderocol susceptibility distributions for Enterobacterales and *Pseudomonas* spp. (**A**,**C**) Disc diffusion inhibition zone distributions for Enterobacterales; (**A**) and *Pseudomonas* spp. (**C**); (**B**,**D**) broth microdilution MIC distributions for Enterobacterales (**B**) and *Pseudomonas* spp. (**D**). Dashed lines indicate EUCAST clinical breakpoints. Bars are color-coded according to susceptibility category: green = susceptible (S), orange = area of technical uncertainty (ATU), red = resistant (R).

Regarding new β-lactam agents, disc diffusion categorized only 37/262 Enterobacterales (14%) as cefiderocol susceptible, while 106/262 (40%) fell within the area of technical uncertainty (ATU) (Figure 2A). Among ATU isolates retested by broth microdilution, 98/106 (92%) were reclassified as susceptible, increasing the susceptibility estimate to 52%. When exclusively assessed by broth microdilution, 224/262 isolates (85%) were categorized as susceptible (Figure 2B). Following ATU resolution, cefiderocol susceptibility rates were 50% for NDM/OXA-48-like co-producers, 49% for NDM-producing isolates, and 60% for VIM producers, with no statistically significant differences between groups (*p* = 0.39). When assessed exclusively by broth microdilution, susceptibility increased to 85%, 88%, and 85%, respectively, and remained comparable across carbapenemase types (*p* = 0.78). Notably, two of four NDM-producing *E. coli* isolates were resistant to cefiderocol, corresponding to a resistance rate of 50% in this subgroup. Supplementary Figure S1 displays the distribution of inhibition zone diameters and MIC results by carbapenemase type.

The categorical agreement between disc diffusion and broth microdilution for cefiderocol was 42%. Discordant results were mainly due to major errors (58%), whereas no very major errors were observed. Most discrepancies corresponded to isolates classified as resistant or within the ATU by disc diffusion that were subsequently categorized as susceptible by broth microdilution. The relationship between inhibition zones diameters and MIC values is shown in Supplementary Figure S2.

**Figure 3:**
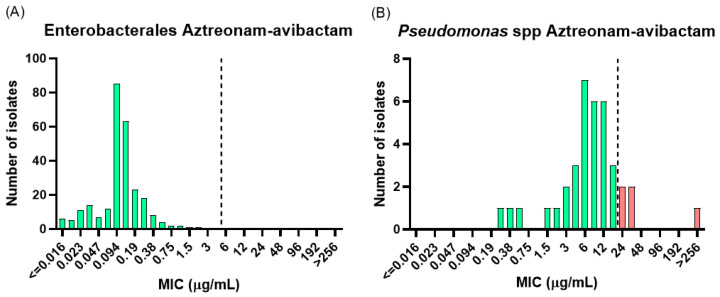
Aztreonam-avibactam gradient strip diffusion MIC distributions for (**A**) Enterobacterales and (**B**) *Pseudomonas* spp. Dashed lines indicate the EUCAST clinical breakpoint for Enterobacterales and the epidemiological cut-off value (ECOFF) for *Pseudomonas* spp., for which no clinical breakpoint is currently defined. Bars are color-coded according to susceptibility category: green = susceptible (S) and red = resistant (R).

Aztreonam-avibactam demonstrated complete in vitro activity against all Enterobacterales isolates (262/262; 100%), with an overall MIC_90_ of 0.38 µg/mL (Figure 3A). The highest MIC observed (2 µg/mL) corresponded to an *E. coli* isolate co-producing NDM and OXA-48-like carbapenemases, remaining within two dilutions of the clinical breakpoint. The MIC and inhibition zone distributions by carbapenemase type are provided in Supplementary Figure S1.

#### 2.2.2. VIM-Producing *Pseudomonas* spp.

All *Pseudomonas* spp. isolates exhibited high levels of multidrug resistance, with meropenem resistance detected in 33/37 isolates (90%). Resistance to aztreonam monotherapy was less frequent (5/37; 14%) and involved *P. aeruginosa* (n = 3), *P. mosselii* (n = 1), and *P. monteilii* (n = 1). No aztreonam resistance was observed among isolates co-producing VIM and GES carbapenemases.

Cefiderocol demonstrated the highest in vitro activity among the agents tested. Only one isolate was categorized as resistant by disc diffusion (Figure 2C). However, subsequent broth microdilution testing yielded a MIC of 0.5 µg/mL, which fell within the EUCAST susceptibility range (Figure 2D).

Aztreonam-avibactam maintained high activity against *Pseudomonas* spp. (Supplementary Figure S3), with only four resistant isolates, all belonging to the subset resistant to aztreonam alone. Notably, only one aztreonam-resistant *P. aeruginosa* isolate exhibited restored susceptibility in the presence of avibactam, with the MIC decreasing from >256 µg/mL to 4 µg/mL (Figure 3B). Overall, the addition of avibactam resulted in limited MIC reduction among aztreonam-resistant *Pseudomonas* spp., suggesting that resistance mechanisms other than β-lactamase activity, such as AmpC hyperproduction or efflux pump overexpression, may play a predominant role.

### 2.3. Temporal Trends

**Figure 4:**
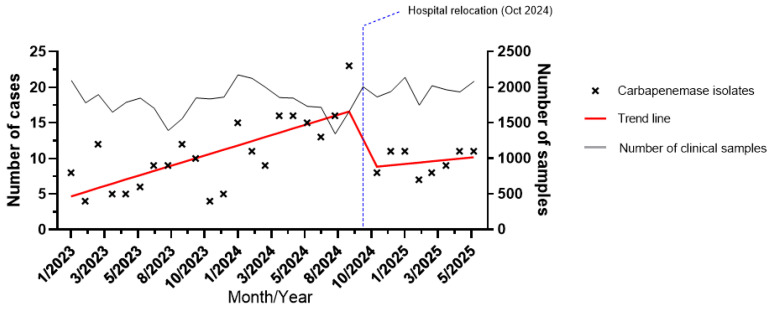
Interrupted time-series analysis assessing the effect of hospital relocation on MBL incidence, January 2023–June 2025. Black crosses represent the number of clinical MBL-producing isolates detected each month, and the red lines depict the pre- and post-relocation regression trends. The vertical dotted blue line marks the relocation of the hospital (October 2024). Data from the relocation month were excluded from the model to avoid misclassification. The grey line represents the total number of clinical samples processed per month.

The temporal analysis was structured around the hospital relocation in October 2024, which was considered a predefined interruption point. The temporal distribution of MBL-producing isolates from January 2023 to June 2025 is shown in Figure 4. A progressive increase in incidence was observed throughout 2023 and 2024, reaching a peak in September 2024 with 23 detected cases. Interrupted time-series analysis showed a statistically significant upward pre-relocation trend, with an increase of 0.6 cases per month (95% CI, 0.36–0.83; *p* < 0.001). This was followed by a marked and statistically significant immediate drop of 7.9 cases (95% CI, −13.6 to −2.1; *p* = 0.009), coinciding with the hospital relocation in October 2024. After relocation, incidence showed a slight upward tendency, although without a statistically significant difference compared with the pre-relocation trend (*p* = 0.43). The monthly volume of processed clinical samples remained stable throughout the study period (*p* = 0.19), indicating that changes in case detection were not attributable to differences in workload. The interrupted time-series analysis model explained approximately half of the observed variability (R^2^ = 0.53), suggesting a moderately strong association between the relocation event and the observed reduction in incidence.

## 3. Discussion

This study provides real-world data of antimicrobial susceptibility patterns among MBL-producing GNB in a large tertiary-care hospital in Madrid, Spain, with a particular focus on the performance of the in vitro activity of cefiderocol and aztreonam-avibactam, and the reliability of different susceptibility testing methods for cefiderocol. Our institution currently faces a significant clinical challenge driven by multidrug-resistant *K. pneumoniae* producing NDM, either alone or in combination with OXA-48-like carbapenemases. This epidemiological profile differs markedly from settings dominated by KPC or OXA-48-like carbapenemases [5,12]. We observed a progressive increase in clinical MBL-producing isolates between 2023 and 2024, largely attributable to NDM-producing *K. pneumoniae* and, to a lesser extent, VIM-producing isolates. This trend is consistent with recent reports from Europe and the United States describing a sustained expansion of NDM-producing Enterobacterales, highlighting the growing clinical relevance of this resistance mechanism [13,14].

The overall susceptibility profile of Enterobacterales in our cohort was strongly influenced by the predominance of *K. pneumoniae* isolates, particularly isolates producing NDM alone or in combination with OXA-48-like carbapenemases, which together accounted for more than two-thirds of the collection. Preliminary molecular typing suggests that these isolates predominantly belong to international high-risk clones ST15, ST147, and ST307. This clonal background was reflected in the antimicrobial resistance phenotype, characterized by high aminoglycoside susceptibility among NDM/OXA-48-like co-producers and widespread resistance to most conventional agents, except for colistin among NDM-producing isolates. In contrast, VIM-producing Enterobacterales exhibited greater species diversity and more heterogeneous susceptibility patterns beyond the β-lactam class. In addition, meropenem resistance was markedly higher among NDM producers (≈90%) than among VIM producers (13%), consistent with the stronger carbapenem-hydrolytic activity of enzymes previously described [15].

The high incidence of MBL-producing Enterobacterales at our institution provided a valuable opportunity to evaluate the in vitro activity of cefiderocol and aztreonam-avibactam under conditions of sustained selective pressure, using a large collection of clinical isolates. Cefiderocol demonstrated substantially lower apparent susceptibility rates when assessed by disc diffusion, even after applying the EUCAST recommended workflow for isolates falling within ATU results [16]. In contrast, susceptibility rates (85%) were markedly higher when all isolates were tested by broth microdilution, underscoring the strong impact of the testing methodology on cefiderocol categorization.

Notably, susceptibility categorization changed for most isolates initially classified as resistant by disc diffusion when retested by broth microdilution. These disc diffusion-based findings are consistent with previous reports from Sweden and the United Kingdom, which described cefiderocol susceptibility rates below 60%, and contrast with data from France reporting susceptibility rates above 80%, more closely aligned with our broth microdilution results [10,17,18]. Such discrepancies have been attributed to methodological differences, including media composition, testing approach (disc vs. broth microdilution), and disc manufacturer [9], highlighting the need for greater standardization of cefiderocol susceptibility testing.

In this context, disc diffusion may serve as an initial phenotypic approach to identify clearly susceptible isolates; however, isolates categorized as resistant or within the ATU require mandatory confirmation by broth microdilution to avoid misclassification. Notably, the low categorical agreement (42%) and high rate of major errors (58%) observed in our study are well outside the acceptable thresholds proposed for antimicrobial susceptibility testing methods, which typically recommend categorical agreement ≥90% and major error rates ≤ 3%. These findings indicate that disc diffusion performs poorly for cefiderocol susceptibility testing in MBL-producing Enterobacterales under our conditions. Importantly, the absence of very major errors supports its use as a preliminary assessment tool, but not as a standalone method for clinical decision-making. From a clinical microbiology perspective, these results reinforce that disc diffusion alone is insufficient for reliable cefiderocol categorization.

Cefiderocol susceptibility was broadly similar across MBL groups in our cohort, contrasting with previous reports describing enhanced activity against VIM-producing isolates [19]. This discrepancy may be related to differences in the distribution of NDM variants, which were not characterized in the present study, as specific variants have been associated with reduced cefiderocol susceptibility [20]. Aztreonam-avibactam demonstrated excellent in vitro activity against MBL-producing Enterobacterales, achieving 100% susceptibility across all carbapenemase types, with MIC values remaining several dilutions below the clinical breakpoint. Slightly higher MICs were observed among *E. cloacae* isolates, likely reflecting the contribution of *Amp*C overexpression as previously described [21]. The highest MIC values for both cefiderocol and aztreonam-avibactam were observed in two NDM-producing *E. coli* isolates. Although the underlying resistance mechanisms are under investigation, similar phenotypes have been linked to alterations in penicillin-binding protein 3 in NDM-producing *E. coli* reported in France and India [17,22].

In contrast to Enterobacterales, cefiderocol was the most active agent against VIM-producing *Pseudomonas* spp. In our collection, only one isolate was initially categorized as resistant by disc diffusion and subsequently reclassified as susceptible by broth microdilution. Although aztreonam-avibactam also exhibited high overall activity, its performance was inferior to that of cefiderocol and largely comparable to aztreonam monotherapy. In this setting, the activity of aztreonam-avibactam against VIM-producing *Pseudomonas* spp. appeared largely driven by the intrinsic activity of aztreonam, with avibactam providing only marginal additional benefit in most isolates. This limited effect is likely attributable to resistance mechanisms such as *Amp*C hyperproduction, efflux pump upregulation (MexAB-OprM), or additional β-lactamases such as PER, which are poorly inhibited by avibactam. However, these mechanisms were not specifically investigated in this study and should, therefore, be interpreted with caution [23,24].

Cefiderocol has demonstrated clinical efficacy in the treatment of severe infections caused by carbapenem-resistant isolates [25,26], and is currently recommended by the IDSA as first-line therapy for infections caused by MBL-producing GNB when susceptibility is confirmed [7]. In contrast, aztreonam-avibactam has demonstrated clinical efficacy primarily against Enterobacterales and is, therefore, recommended exclusively for MBL-producing Enterobacterales infections. In high-NDM-prevalence settings, our findings provide microbiological support for the potential use of aztreonam-avibactam when MBL-producing Enterobacterales are suspected; however, this interpretation should be made with caution, as our study is based on in vitro data and does not include clinical outcome analyses. Similarly, cefiderocol may be considered as a potential option for empirical coverage of MBL-producing *Pseudomonas* spp. Given this epidemiological situation, the clinical microbiology laboratory plays a fundamental role in the recommendation and appropriateness of empirical or targeted antibiotic treatment with cefiderocol and aztreonam/avibactam in patients with suspected or confirmed infections against MBL-producing GNB.

Finally, we evaluated the temporal trends of MBL-producing infections in relation to hospital relocation. Although a clear temporal association was observed between relocation and a reduction in MBL-related infections, this study was not designed to establish causality. Potential contributing factors, including infrastructural or environmental changes, were not specifically assessed and, therefore, cannot be inferred from our data. The observed reduction should be interpreted as a descriptive finding, and further studies incorporating infection control and epidemiological variables would be required to better understand the underlying drivers. Continued surveillance will be essential to monitor future trends.

Our study has limitations. First, it was conducted at a single tertiary-care hospital, and, therefore, the local epidemiology, particularly the predominance of NDM and NDM/OXA-48-like-producing *K. pneumoniae*, may not reflect other settings. However, the high incidence of these organisms offered a unique opportunity to evaluate their behavior under intense selective pressure and to increase the relevance of our findings to real-world decision making. Second, for aztreonam-avibactam in *Pseudomonas* spp., interpretation was based on ECOFFs in the absence of EUCAST clinical breakpoints, in line with current EUCAST recommendations, which should be considered when interpreting these results. Third, molecular analyses were restricted to carbapenemase genes; additional mechanisms, such as porin loss, efflux pump upregulation, and siderophore receptor mutation, were not systematically assessed. Future sequencing-based studies are needed to better elucidate the determinants of reduced susceptibility.

## 4. Materials and Methods

### 4.1. Bacterial Isolates

This study was conducted at Hospital Universitario 12 de Octubre (Madrid, Spain), a 1300-bed tertiary-care teaching hospital accredited by the Spanish National Accreditation Body (ENAC) under the UNE-EN ISO 15189 standard [27], serving a catchment population of approximately 460,000 inhabitants (≈44,000 admissions/year).

We included MBL-producing clinical isolates recovered from patients with a documented infection. Enterobacterales were collected between January 2023 and June 2025, whereas the collection period for MBL-producing *Pseudomonas* spp. extended from January 2022 to June 2025 in order to obtain a representative number of isolates, which are considerably less frequent in our institution. Non-duplicated clinical isolates were obtained from blood, urine, respiratory, abdominal, and skin/soft-tissue samples from infected patients. Screening and colonization isolates were excluded based on clinical request forms and laboratory information system records. A total of 320 isolates were collected, of which 299 were available for antimicrobial susceptibility testing.

### 4.2. Antimicrobial Susceptibility Testing and Molecular Characterization

Bacterial identification was performed from colonies grown on blood agar plates using matrix-assisted laser desorption/ionization time-of-flight mass spectrometry (MALDI-TOF MS; Bruker Daltonics, Bremen, Germany). Antimicrobial susceptibility testing was conducted by microdilution using a semi-automated system (MicroScan, Beckman Coulter Diagnostics, Brea, CA, USA; Neg MIC Panel 63), including piperacillin-tazobactam, ceftriaxone, ceftazidime, cefepime, aztreonam, imipenem, meropenem, gentamicin, tobramycin, amikacin, ciprofloxacin, and trimethoprim-sulfamethoxazole. Cefiderocol susceptibility was evaluated by both disc diffusion (30 µg discs, Liofilchem, Roseto degli Abruzzi, Italy) on Mueller–Hinton agar (bioMérieux, Marcy-l’Étoile, France) [28] and broth microdilution using UMIC strips (Bruker Daltonics, Bremen, Germany), which incorporate iron-depleted conditions in accordance with EUCAST recommendations and have been previously evaluated for cefiderocol susceptibility testing [9]. Results were interpreted using three approaches: (i) disc diffusion results as obtained, (ii) disc diffusion results after resolution of isolates falling within the ATU, which were systematically retested and categorized based on broth microdilution results, and (iii) results obtained exclusively by broth microdilution. Aztreonam-avibactam Minimum Inhibitory Concentrations (MICs) were determined by gradient diffusion (Etest, bioMérieux, Marcy-L’Etoile, France). EUCAST clinical breakpoints (version 15.0, 2025) [29] were applied for all antimicrobial susceptibility testing results. For aztreonam-avibactam against *Pseudomonas* spp., where no clinical breakpoints are currently defined, epidemiological cut-off values (ECOFFs) were used for interpretation [30].

In Enterobacterales, screening for carbapenemase production was based on reduced carbapenem susceptibility (ertapenem/meropenem MIC > 0.125 mg/L), whereas in *Pseudomonas* spp., ceftolozane-tazobactam resistance was used as a surrogate marker [31]. Carbapenemase detection was initially performed using immunochromatography (NG-Test^®^ CARBA 5; NG Biotech, Guipry, France), followed by molecular confirmation. Multiplex PCR was carried out using the Allplex™ Entero-DR Assay (Seegene Inc., Seoul, Republic of Korea) on a CFX96™ real-time PCR system (Bio-Rad Laboratories, Hercules, CA, USA), targeting *bla*_KPC_, *bla*_NDM_, *bla*_VIM_, *bla*_IMP_, *bla*_OXA-48_-like, and *bla*_CTX-M_ genes. Additional *bla*_GES_ screening was performed by real-time PCR (LightCycler 2.0, Roche, Basel, Switzerland), followed by Sanger sequencing (ABI prism 3100 DNA Sequencer; Applied Biosystems, Norwalk, CT, USA) [32].

Antimicrobial susceptibility testing quality control was performed using *E. coli* ATCC 25922, *P. aeruginosa* ATCC 27853, *K. pneumoniae* ATCC 700603, and BAA-2146, according to EUCAST recommendations.

### 4.3. Statistical Analysis

Statistical analyses and figure generation were performed using GraphPad Prism v 8.0.2 (GraphPad Software, San Diego, CA, USA) and RStudio v.2023.06.1 (R Foundation for Statistical Computing, Vienna, Austria). MIC_50_ and MIC_90_ values were calculated for each species–antibiotic combination. For cefiderocol, categorical agreement (CA), major errors (MEs), and very major errors (VMEs) between disc diffusion and broth microdilution were assessed according to standard definitions. Comparisons of categorical variables between groups were performed using the chi-square test. Confidence intervals (95% CI) were calculated where appropriate.

During the study period, our hospital underwent a complete relocation. Given this major institutional change, an interrupted time-series analysis was performed to explore temporal trends in MBL-producing infections before and after hospital relocation. Temporal trends in MBL-producing isolates were evaluated using interrupted time-series analysis with segmented multiple linear regression. The model included baseline trend, immediate level change, and post-intervention trend following hospital relocation in October 2024. The relocation month was excluded from the model to prevent transition-related misclassification. A two-sided *p*-value < 0.05 was considered statistically significant.

## 5. Conclusions

This study provides an updated evaluation of the in vitro activity of cefiderocol and aztreonam-avibactam against a large collection of MBL-producing GNB recovered from clinical samples in a tertiary-care hospital. Cefiderocol susceptibility was substantially underestimated when assessed by disc diffusion, underscoring the importance of confirming isolates categorized as resistant or within the area of technical uncertainty by broth microdilution.

Aztreonam-avibactam demonstrated consistently high in vitro activity against MBL-producing Enterobacterales, whereas cefiderocol performance was highly dependent on the susceptibility testing method used. These findings support a differentiated role for both agents according to the infecting pathogen and resistance mechanism, with cefiderocol being particularly relevant for infections caused by VIM-producing *Pseudomonas* spp. Confirmation by broth microdilution is essential to avoid underestimation of cefiderocol susceptibility.

A significant reduction in MBL-related infections was observed following hospital relocation, suggesting that infrastructural and environmental factors may influence transmission dynamics, although causality cannot be established. Overall, these results highlight the importance of robust susceptibility testing strategies to support antimicrobial resistance surveillance and stewardship in high-risk healthcare settings.

## Figures and Tables

**Table 1:** MIC_50_/MIC_90_ values (mg/L) of MBL-producing GNB (n = 299) for selected antimicrobial agents. Data are presented overall and stratified by bacterial species and carbapenemase type. Grey-shaded cells indicate antimicrobial-organism combinations for which the MIC_50_ value was below the EUCAST clinical breakpoint.

Antimicrobial Agent	All MBL Enterobacterales (n = 262)	*K. pneumoniae* NDM + OXA-48 (n = 111)	*K. pneumoniae* NDM (n = 84)	Enterobacterales VIM (n = 55)	*E. cloacae* VIM (n = 16)	*Pseudomonas* spp. VIM (n = 37)
Piperacillin-tazobactam	>16/>16	>16/>16	>16/>16	>16/>16	>16/>16	>16/>16
Ceftriaxone	>16/>16	>16/>16	>16/>16	>16/>16	>16/>16	-
Ceftazidime	>32/>32	>32/>32	>32/>32	>32/>32	>32/>32	>32/>32
Cefepime	>8/>8	>8/>8	>8/>8	>8/>8	>8/>8	>8/>8
Aztreonam	>4/>4	>4/>4	>4/>4	>4/>4	>4/>4	8/64
Imipenem	>4/>4	>4/>4	>4/>4	>4/>4	>4/>4	>4/>8
Meropenem	>8/>8	>8/>8	>8/>8	2/>8	3/>8	>8/>8
Aztreonam-avibactam	0.094/0.38	0.125/0.38	0.094/0.125	0.032/0.5	0.094/1	8/24
Cefiderocol	1/4	1/4	0.5/8	1/4	1/4	0.25/1
Gentamicin	≤2/>4	≤2/>4	>4/>4	>4/>4	>4/>4	>4/>4
Tobramycin	>4/>4	>4/>4	>4/>4	>4/>4	>4/>4	>4/>4
Amikacin	≤8/>16	≤8/16	16/>16	≤8/≤8	≤8/≤8	16/>16
Ciprofloxacin	>1/>1	>1/>1	>1/>1	1/>1	>1/>1	>1/>1
Trimethoprim-sulfamethoxazole	>4–76/>4–76	>4–76/>4–76	>4–76/>4–76	>4–76/>4–76	>4–76/>4–76	>4–76/>4–76
Colistin	≤2/≤2	≤2/≤2	≤2/≤2	≤2/>4	≤2/≤2	≤2/≤2

## Data Availability

The original contributions presented in this study are included in the article/Supplementary Material. Further inquiries can be directed to the corresponding author.

## Supplementary Materials

The following supporting information can be downloaded at: https://www.mdpi.com/article/10.3390/antibiotics15040380/s1, Figure S1: Cefiderocol and aztreonam-avibactam susceptibility profiles in MBL-producing Enterobacterales subgroups; Figure S2: Categorical agreement between cefiderocol disc diffusion and broth microdilution MIC results for Enterobacterales; Figure S3: Comparison of aztreonam (AZT) and aztreonam-avibactam (AZA) MICs among VIM-producing *Pseudomonas* spp.

## Abbreviations

The following abbreviations are used in this manuscript:

MBL	Metallo-β-Lactamase
EUCAST	European Committee on Antimicrobial Susceptibility Testing
GNB	Gram-Negative Bacilli
WHO	World Health Organization
IDSA	Infectious Diseases Society of America
ENAC	Spanish National Accreditation Body
ECOFF	Epidemiological Cut-Off Value
CA	Categorical Agreement
ME	Major Error
VME	Very Major Error
