# Supplementary material for: Impact of Susceptibility Testing Methodology on the Positioning of Cefiderocol and Aztreonam-Avibactam Against Metallo-β-Lactamase-Producing Gram-Negative Bacteria
Source: Antibiotics (Basel). 2026 Apr 9;15(4):380. doi: 10.3390/antibiotics15040380 (PMC13113646; doi:10.3390/antibiotics15040380)
Supplement: Supplementary file 1 [file antibiotics-15-00380-s001.zip › antibiotics-4224445-supplementary.pdf]

Supplementary Material

Title: Impact of susceptibility testing methodology on the positioning of cefiderocol and aztreonam-avibactam against metallo-β-lactamase producing Gram-negative bacteria

Authors: Fernando del Nogal-Labrador, Beatriz González-Blanco, María Isabel Sanz, Raúl Recio, Patricia Brañas, Irene Muñoz-Gallego, Esther Viedma, Jennifer Villa.

Journal: Antibiotics

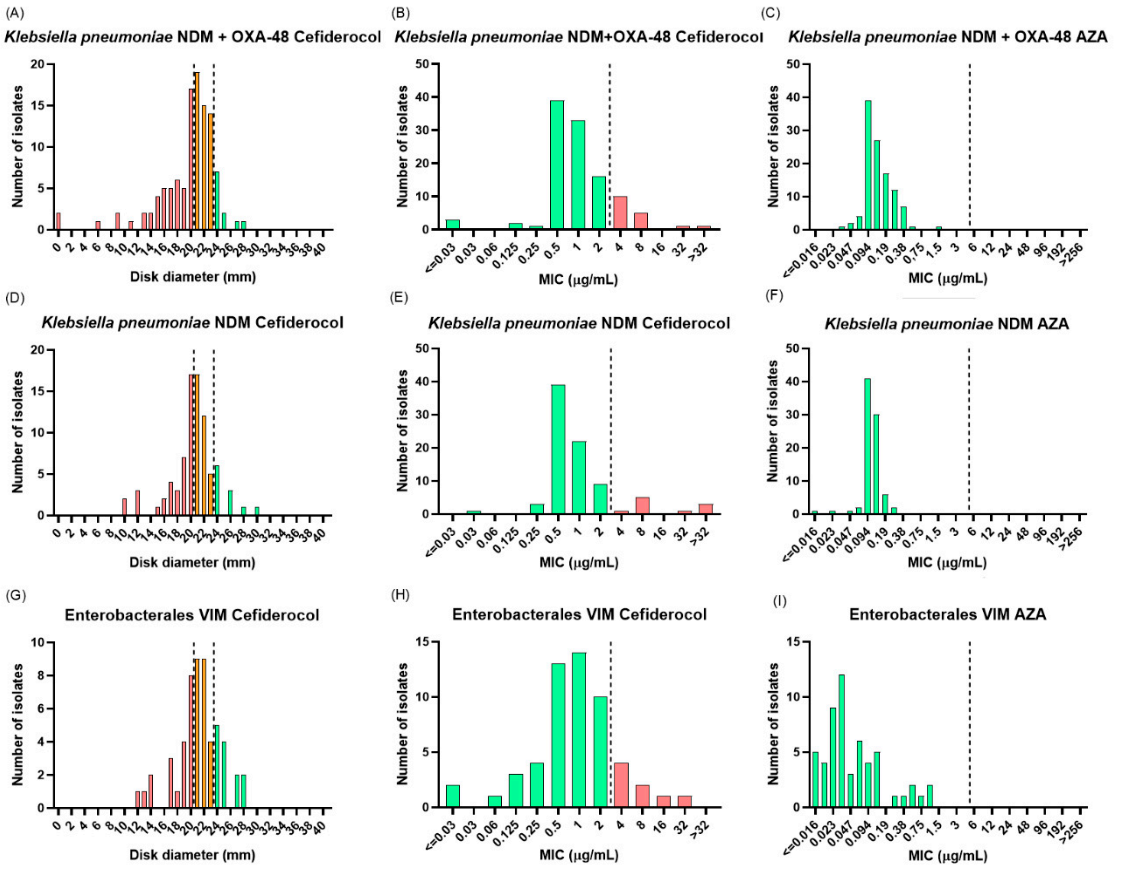

Supplementary Figure S1

Cefiderocol and aztreonam-avibactam susceptibility profiles in MBL-producing Enterobacterales subgroups.

Susceptibility distributions for *K. pneumoniae* co-producing NDM/OXA-48-like (A-C), *K. pneumoniae* producing NDM (D-F), and VIM-producing Enterobacterales (G-I). Panels display cefiderocol disc diffusion results (A, D, G), cefiderocol MICs determined by broth microdilution (B, E, H), and aztreonam-avibactam MICs determined by gradient strip diffusion (C, F, I).

Dashed lines represent EUCAST clinical breakpoints. Bars are color-coded to indicate

susceptibility categories: green = susceptible (S), orange = area of technical uncertainty (ATU), red = resistant (R).

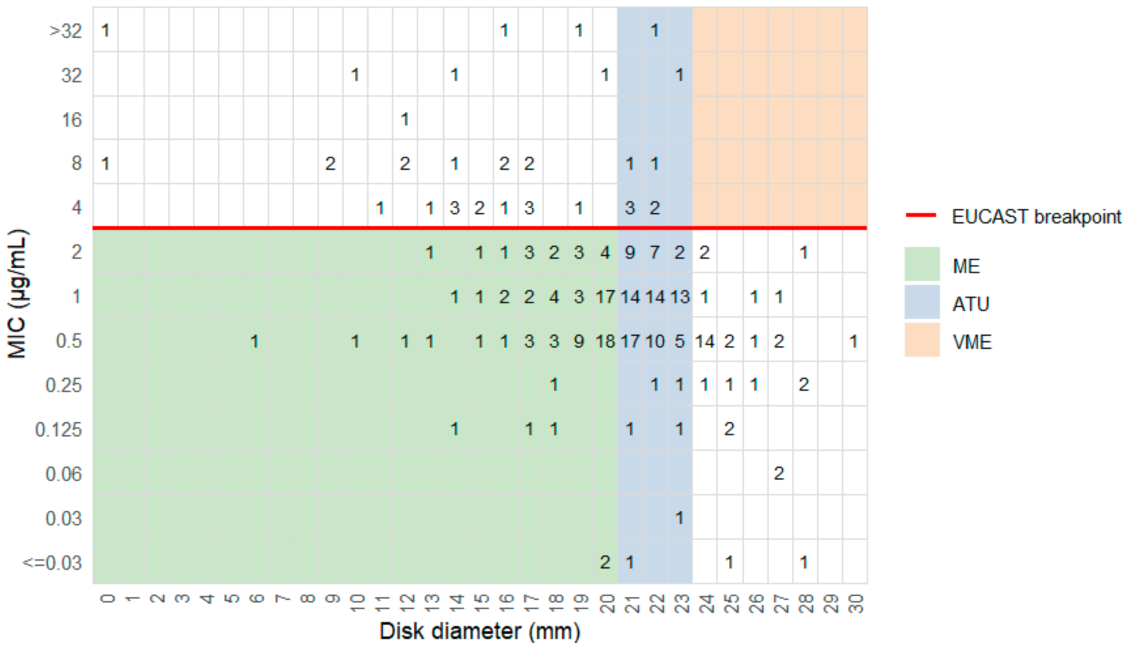

Supplementary Figure S2

Categorical agreement between cefiderocol disc diffusion and broth microdilution MIC results for Enterobacterales (n = 262).

Scatterplot displaying the relationship between inhibition zone diameter (x-axis) and MIC values (y-axis). The horizontal red line indicates the EUCAST clinical breakpoint (MIC ≤2 µg/mL), and the shaded vertical area represents the EUCAST area of technical uncertainty (ATU, 20-21 mm). Data points are colour-coded according to categorical interpretation relative to the reference method (broth microdilution): green = major errors (ME), blue = ATU, orange = very major errors (VME). Numbers within cells represent isolate counts.

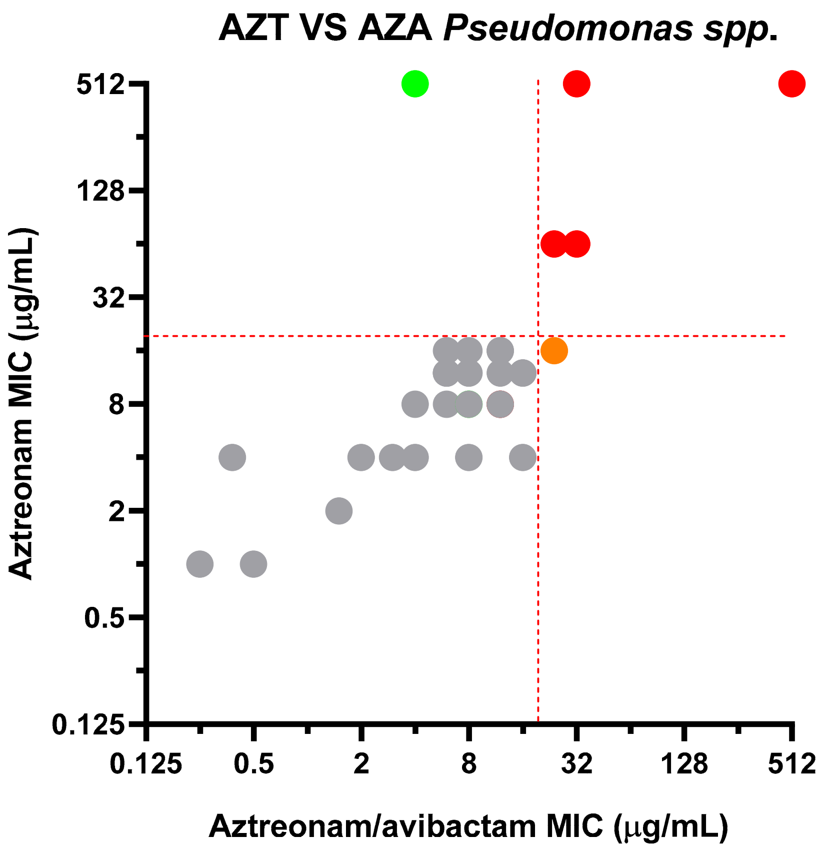

Supplementary Figure S3

Comparison of aztreonam (AZT) and aztreonam-avibactam (AZA) MICs among VIM-producing Pseudomonas spp: Each point represents an individual isolate. Colours reflect the change in categorical interpretation (susceptible/resistant) when comparing aztreonam alone with the combination: grey indicates isolates that were susceptible to both AZT and AZA; green indicates isolates that were resistant to AZT but became susceptible with AZA, reflecting restored susceptibility; red indicates isolates resistant to both agents, showing no restoration; and orange corresponds to isolates that were susceptible to AZT but resistant to AZA, representing an atypical phenotype. The horizontal red dashed line represents the EUCAST clinical breakpoint for aztreonam, while the vertical red dashed line corresponds to the ECOFF for aztreonam-avibactam, as no clinical breakpoint is currently defined for this combination in Pseudomonas spp.
